# Probing Neural Transplant Networks* In Vivo* with Optogenetics and Optogenetic fMRI

**DOI:** 10.1155/2016/8612751

**Published:** 2016-05-12

**Authors:** Andrew J. Weitz, Jin Hyung Lee

**Affiliations:** ^1^Department of Bioengineering, Stanford University, Stanford, CA 94305, USA; ^2^Department of Neurology and Neurological Sciences, Stanford University, Stanford, CA 94305, USA; ^3^Department of Neurosurgery, Stanford University, Stanford, CA 94305, USA; ^4^Department of Electrical Engineering, Stanford University, CA 94305, USA

## Abstract

Understanding how stem cell-derived neurons functionally integrate into the brain upon transplantation has been a long sought-after goal of regenerative medicine. However, methodological limitations have stood as a barrier, preventing key insight into this fundamental problem. A recently developed technology, termed optogenetic functional magnetic resonance imaging (ofMRI), offers a possible solution. By combining targeted activation of transplanted neurons with large-scale, noninvasive measurements of brain activity, ofMRI can directly visualize the effect of engrafted neurons firing on downstream regions. Importantly, this tool can be used to identify not only whether transplanted neurons have functionally integrated into the brain, but also which regions they influence and how. Furthermore, the precise control afforded over activation enables the input-output properties of engrafted neurons to be systematically studied. This review summarizes the efforts in stem cell biology and neuroimaging that made this development possible and outlines its potential applications for improving and optimizing stem cell-based therapies in the future.

## 1. Introduction

Transplantation of stem cell-derived neurons is a promising therapeutic strategy for a diverse spectrum of neurological disorders, including Parkinson's disease, amyotrophic lateral sclerosis, and stroke [1]. A shared feature of these pathological conditions is damage to or complete loss of endogenous neuronal populations and circuits. Transplantation of stem cell-derived neurons has been shown to restore normal network properties through synaptic integration and even behavioral recovery across species and disease models [2–4]. However, the extent to which recovery is driven by cell replacement and neural circuitry repair compared to other mechanisms (e.g., growth factor secretion, immunomodulation, or remyelination) remains controversial. Resolving this issue will be vital for translating stem cell therapies from the laboratory to clinical applications, but addressing it head-on has been challenging due to technical limitations. In particular, traditional methods and imaging techniques have been unable to test the causal relationship between an engrafted population's electrical activity and markers of functional recovery [5]. Fortunately, with the rapid development of optogenetic technologies over the last decade [6, 7], scientists are now able to control the electrical activity of neuronal populations with remarkable spatial and temporal precision. The inevitable integration of optogenetic tools with stem cell-derived populations has since enabled selective excitation and/or inhibition of stem cell-derived tissue and the causal interrogation of graft-host relationships both* in vitro* and* in vivo* [8–15]. These studies have laid the foundations for fundamentally new and improved characterizations of functional integration.

Several studies combining optogenetic technologies with neuronal transplants have already demonstrated the necessity and importance of neural circuit repair in driving functional improvements following stroke or 6-OHDA treatment in rats [12, 13]. Understanding the exact circuit mechanisms underlying these improvements and similar ones yet to be discovered will be vital for optimizing stem cell based therapies. Questions of interest include which efferent projections of the neural graft are necessary and/or sufficient for functional recovery and which temporal patterns of graft activity facilitate recovery. Ultimately, these issues are related to the broader challenge of characterizing the dynamic and causal influence of graft activity on downstream regions of the brain. A key area of research moving forward will thus be the detailed study of regenerated neural circuits and their functional relationship with host networks. This issue can be partially addressed by combining optogenetic control of graft function with electrophysiology recordings, a technique that has been successfully used to demonstrate graft-host, graft-graft, and host-graft interactions* in vitro* [8–11]. However, for a broader and more complete investigation of graft-host circuit function, we need a tool that can evaluate the response to graft modulation with regional specificity across the whole intact brain.

In response to this need, our research group recently developed a platform for visualizing whole-brain activity during selective control of engrafted neurons using functional MRI [16]. As a proof of principle that selective excitation of engrafted neurons could be combined with whole-brain functional imaging to identify graft-driven activity at local and downstream regions, we transplanted neurons derived from human induced pluripotent stem cells (iPSCs) or embryonic stem cells (hESCs) to the striatum of immunosuppressed rats. Following functional integration, targeted stimulation of the graft resulted in positive fMRI signals both locally and at remote regions such as the cingulate cortex, septum, and hippocampus. Remarkably, these changes in the fMRI signal were associated with corresponding increases in neuronal firing. Through these experiments, we demonstrated that ofMRI-based neuroimaging can be used to visualize the whole-brain effect of precise changes in an engrafted population's activity. Such a method confers several advantages over electrophysiology-based techniques. First, rather than restricting the investigation to only a handful of downstream regions, activity across the whole-brain can be measured simultaneously. This is especially important for identifying graft-driven activity in regions that may not be connected directly with the transplanted population. Second, optogenetic fMRI (ofMRI) can be performed* in vivo* and is relatively noninvasive in nature. This feature enables longitudinal studies within the same subject, which can be used to characterize the progress of functional integration over time.

## 2. Optogenetic Control of Stem Cells

Optogenetics has revolutionized the field of neuroscience by providing researchers with a toolbox to perturb the electrical activity of specific cell types within the intact brain. Under this paradigm, neurons are genetically engineered using lentiviral, adenoviral, or transgenic techniques to express light-sensitive transmembrane proteins, known as opsins. When these opsins are illuminated with a certain wavelength of light, they undergo a conformational change and allow charged ions to cross the cellular membrane, thereby inducing a change in transmembrane potential. Importantly, transduced neurons can be controlled in awake, behaving animals using implanted light delivery devices, such as optical fibers or micro-LEDs [17, 18]. The first opsin to achieve widespread use, channelrhodopsin-2 (ChR2), is excitatory in nature (i.e., illumination causes membrane depolarization) and can be used to drive action potentials with millisecond temporal resolution [19, 20]. While ChR2 is often sufficient to investigate the causal role of a particular population in driving behavior or downstream changes in neural activity, the optogenetic toolbox is also constantly expanding to enable more sophisticated modes of neural circuit interrogation. For example, inhibitory opsins like the inward chloride pumps halorhodopsin [21, 22] and Jaws [23] or the outward proton pump archaerhodopsin [24] can be used to selectively silence neurons via hyperpolarization. The kinetic profile of opsins has also expanded to enable prolonged subthreshold depolarization or hyperpolarization with a single light pulse [25, 26]. In addition, the development of new targeting strategies with different promoters and Cre-lox systems has diversified the specific types of neurons (and excitable tissue in general) that can be controlled optically [17, 27]. Stem cell-derived neurons have been a particularly sought-after target of optogenetic manipulation, with the goal of controlling neural graft activity* in vivo*.

Weick et al. [8] were the first to demonstrate optical control of stem cell-derived neurons. Human embryonic stem cells were engineered to express ChR2 using the pan-neuronal* synapsin-1* promoter with lentiviral transfection.* In vitro*, neurons could reliably fire action potentials in response to light pulses delivered at frequencies up to 20 Hz. Moreover, it was shown that excitatory and inhibitory postsynaptic potentials could be evoked in cocultured non-ChR2-expressing neurons. Following ventricular transplantation in severe combined immunodeficient (SCID) mouse pups, the ChR2-expressing hESC-derived neurons successfully survived for up to six months. This allowed the graft-host network properties to be analyzed in acute brain slices* ex vivo*. In particular, stimulation of engrafted neurons resulted in delayed postsynaptic currents in putative host neurons. A follow-up study by the same group showed that optical stimulation of ChR2-expressing hESC-derived neurons that were transplanted to mouse CA3 could modulate host pyramidal neurons and network excitability in acute slices* ex vivo* as well [9]. This work set the stage for future optogenetic interrogations of graft-host networks, demonstrating for the first time a causal relationship between graft electrical activity and the host's neuronal response.

Work by Tønnesen et al. [10] built upon these studies by using optogenetic excitation and inhibition to characterize bidirectional graft-host synaptic interactions. Stem cell-derived dopaminergic neurons, transfected with ChR2 using the excitatory CaMKIIa promoter and a lentiviral construct, were transplanted into organotypic cultures of wild type mouse striatum. Similar to the above studies, optical excitation of the transplanted neurons elicited postsynaptic currents in host neurons. However, responses to optical stimulation could also be measured in non-ChR2-expressing graft neurons, demonstrating the ability to characterize causal graft-graft interactions as well. Moreover, in separate preparations, optogenetic control of host neurons was used to characterize causal host-graft relationships. Interestingly, optical silencing of host neurons resulted in increased activity of the engrafted population, while optical excitation of host neurons evoked no discernible change in the graft population. These experiments highlight the ability of optogenetics to dissect the complex interactions between transplanted stem cell-derived neurons and host networks in the central nervous system. However, a key limitation that they suffer from is a restriction to* in vitro* or* ex vivo* analyses. More recent work has overcome this barrier, enabling targeted control of transplanted stem cell-derived neurons* in vivo*.

Perhaps unsurprisingly, the first study to demonstrate optical control of stem cell-derived neurons* in vivo* was performed in the peripheral nervous system [14]. Motor neurons derived from embryonic stem cells were transfected with ChR2 using the pan-cellular CAG promoter and engrafted into a denervated peripheral nerve of adult mice. Following sufficient time to allow for functional integration, optical stimulation delivered to the nerve resulted in sustained contraction of the associated muscle in anesthetized animals. While this experiment demonstrated an important result in the context of restoring lost functionality via neural circuit replacement, the relationship between functional graft-host integration and restoration of function in the central nervous system is much more complex. For example, restoring normal motor control in hyper- and hypokinetic disorders or cognitive performance in conditions like Alzheimer's disease and stroke may require a complex interaction of excitatory, inhibitory, and neuromodulatory influences between the graft and different endogenous populations.

In support of this, more recent studies have shown causal, complex relationships between the electrical activity of engrafted stem cell-derived neurons and functional recovery in the central nervous system. In the work by Steinbeck et al. [13], for example, hESCs were transduced to express the inhibitory opsin halorhodopsin and differentiated into mesencephalic dopaminergic (mesDA) neurons. Following 6-OHDA lesions in adult SCID mice, the hESC-derived mesDA neurons were transplanted to striatum. Animals grafted with the mesDA neurons exhibited a complete recovery in the corridor behavioral test (which tests for motor asymmetries) ~4 months after transplantation. Importantly, this recovery was reversed by optically silencing the engrafted population during the test. In another series of experiments by Daadi et al. [12], ChR2-expressing neural stem cells were transplanted to the striatum of ischemic lesioned adult rats. Animals that then received chronic excitatory stimulation over the course of 4 weeks exhibited enhanced sensorimotor performance and forelimb use compared to nonstimulated controls. Notably, gene expression analysis revealed significant upregulation and downregulation of various transcripts, suggesting that the improved performance could also result from mechanisms other than cell replacement. These two studies complement one another by reinforcing the hypothesis that functional integration in the central nervous system acts in complex ways. While, in one study, functional recovery was temporarily reversed by acute inhibition of the engrafted neurons, in the other, functional recovery was improved by long-term stimulation of the transplanted cells. Both studies indicate that the electrical firing pattern of transplanted cells may be critical for driving recovery. To shed light on this process, future studies will need to characterize the causal influence of graft electrical activity on other neural circuits. In the following section, we summarize the development of ofMRI and its application to address this problem via noninvasive measurements of whole-brain activity during selective perturbations of graft cells.

## 3. Optogenetic fMRI

Functional MRI is a neuroimaging technique that detects local changes in oxygenation levels in the vasculature of the brain [28]. Because neurons that are electrically active have a higher metabolism and therefore greater energy demand, the blood-oxygen-level-dependent (BOLD) signal that is measured with fMRI can be used as an indirect measure of neural activity [29]. Over the last several decades, fMRI has achieved widespread use in the neuroscience community, for both clinical applications and basic research [30]. Key advantages of fMRI include its noninvasive nature, independence from radioactivity tracers, and large, whole-brain field of view. Furthermore, novel developments in MR technology are constantly enabling greater spatial and temporal resolution [22, 31].

In the first demonstration that fMRI could be combined with simultaneous optogenetic stimulation [32], it was shown that targeted excitation of excitatory neurons in motor cortex and thalamus drives robust increases in the BOLD signal both locally at the site of stimulation and at downstream, monosynaptically connected regions. The observation of remotely evoked BOLD signals was an especially important finding, because it signalled that ofMRI can be used more broadly as a general brain mapping tool for measuring dynamic brain function [33–35]. Since this initial report, a number of studies have utilized the ofMRI toolbox to characterize the functional connectivity of various other brain regions, including somatosensory cortex, medial prefrontal cortex, hippocampus, and ventral tegmental area [36–43]. Advances in electrode design have also made it possible to simultaneously record neural activity during ofMRI experiments without artifacts in the electrical recordings or images [44].

The development of a combined ofMRI-stem cell technology introduces several new challenges that come with* in vivo* stimulation of transplanted neurons. First, stable and uniform opsin expression may not be easily achieved in the population of interest. For example, the inherent variability in transduction efficacy associated with viral infection of hESC-derived neurons led one group to develop a clonal ChR2-expressing hESC line [8]. Second, the number of neurons activated by stimulation is potentially much less than that which would be activated if an endogenous population was targeted. Transplanted cells that grow into mature neurons will potentially be limited in number, which can lead to a relatively low functional signal-to-noise ratio (SNR). Another related concern is that engrafted neurons may migrate over time and move away from the light delivery location, further reducing the number of mature, transplanted neurons that are stimulated. A fourth point that must be considered is the change in local vasculature that may occur at the transplantation site as the graft becomes vascularized. While evidence suggests that optical stimulation itself does not alter blood flow in the brain [45], vascularization of the graft can potentially have confounding effects on the locally evoked BOLD signal, establishing the need for control experiments to ensure that any local signal results directly from stimulation of the opsin-expressing neural transplant.

Overcoming these concerns, our research group developed a series of methods and conducted experiments demonstrating that ofMRI is a feasible tool for visualizing the influence of precisely controlled neural transplant firing on whole-brain networks in an intact animal [16]. After transplanting ChR2-expressing human iPSC- or hESC-derived neurons to rat striatum, we found that engrafted neurons had structurally integrated with the host brain, sending axonal projections via white matter tracts to remote regions such as the cingulate cortex, septal nuclei, and hippocampus. To relate this structural integration with whole-brain activity causally driven by graft firing, we next performed whole-brain fMRI during selective stimulation of the engrafted population. fMRI activations were detected by identifying voxels significantly modulated to repeated 20-second periods of stimulation. To minimize SNR loss and increase sensitivity of detection (an important consideration given the SNR issues described above), a highly accurate Gauss-Newton motion correction algorithm was applied [46]. Both locally at the site of stimulation and at downstream regions where axonal projections were observed, robust positive BOLD signals were detected. Confirming that these changes reflected underlying neuronal activity, single-unit recordings at these regions also revealed stimulation-induced increases in spike activity. Importantly, stimulation with off-spectrum light (i.e., a wavelength that does not activate ChR2) failed to drive BOLD signals in regions that were modulated by on-spectrum 493 nm light delivery. In addition, similar results were obtained when either iPSCs or hESCs were transplanted, suggesting that ofMRI can serve as a general platform for the stem cell biology community. Finally, it is important to note that while the fMRI signals observed in these experiments were generally correlated with anatomical projections emanating from the graft, other ofMRI studies have reported the detection of fMRI activity in regions not directly connected to the directly modulated population [47]. Thus, using ofMRI, it will in principle be possible to detect activity across multiple synapses from the graft and delineate entire graft-host functional circuits.

## 4. Future Directions and Conclusion

**Figure 1:**
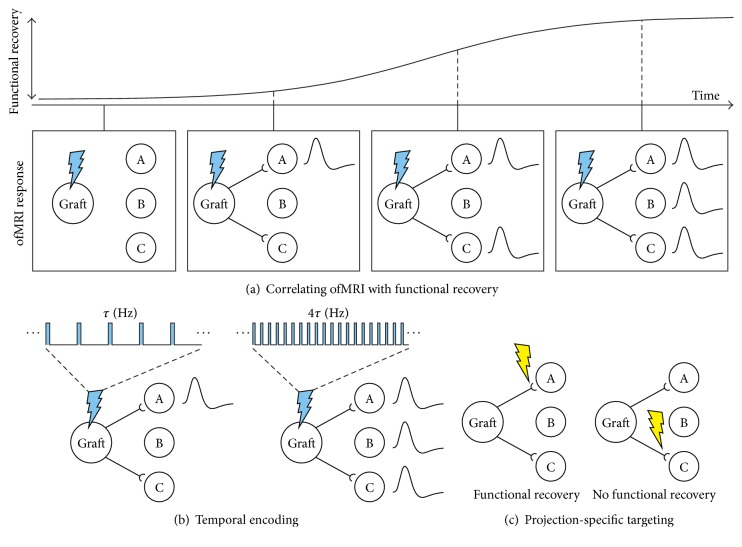
Optogenetic control of neuronal grafts and its combination with fMRI can be used to characterize dynamic graft-host interactions and shed light on neuronal circuit mechanisms underlying functional recovery. (a) The noninvasive nature of ofMRI allows longitudinal studies to be performed in parallel with behavioral assessments of functional recovery. This cartoon illustrates an example in which stimulation of engrafted neurons evokes activity in more downstream regions (represented as nodes A, B, and C) over time. Hemodynamic response functions are shown as insets to indicate the presence of fMRI signal modulations. These functional connections can be matched to behavioral readouts, providing information on which graft-host brain networks underlie functional recovery. In this example, the graft exhibits structural integration and sends projections to two remote regions (nodes A and C). However, the time courses over which these projections influence network activity can differ. Eventually, stimulation can even result in the modulation of a region that does not receive direct projections from the graft itself (node B) but is influenced polysynaptically by the graft-host-host network. (b) Similar to endogenous neuronal populations, engrafted neurons may exert different effects on downstream brain regions depending on the specific temporal pattern of their firing. ofMRI enables precise temporal patterns to be played into the graft while imaging whole-brain activity. This cartoon illustrates an example in which the frequency of stimulation causes the engrafted neurons to modulate the activity of additional downstream regions when stimulation frequency is increased. (c) Projection-specific targeting enables the role of particular graft-host projections to be studied. In this cartoon, for example, functional recovery occurs when the graft-to-A projection in inhibited but does not occur when the graft-to-B projection is inhibited. Experimental paradigms like this can be used to infer which projections are necessary for functional recovery.

The recently developed ofMRI-stem cell imaging platform is expected to play a pivotal role in the future development and optimization of stem cell-based therapies for neurological disorders. Central to this work will be imaging graft-driven changes in brain activity following transplantation to a specific animal model of disease (e.g., stroke and Parkinson's disease). Because ofMRI experiments do not require any invasive procedure beyond the initial transplantation and implantation of a light delivery device, this imaging-based approach can also be used in parallel with longitudinal studies that monitor functional behavior recovery over time. Under this paradigm, improvements in behavior can be correlated with specific changes in the causal function of graft-host circuitry within the same animal (Figure 1(a)). Longitudinal ofMRI experiments may therefore provide key insight into the underlying changes and replacement of neuronal circuitry that mediate behavioral improvement.

Optogenetic control of neural grafts can also be applied to identify the influence of different temporal firing patterns on functional recovery or to characterize the functional, dynamic properties of graft-host connections in general. For example, we [47] and others [48, 49] have discovered that different frequencies of optogenetic stimulation in the naïve brain can affect behavior or influence how the targeted brain structure interacts with remote regions. Similarly, while targeted graft stimulation with one temporal paradigm may result in significant functional recovery [12], stimulation with another may worsen or improve this outcome. Using ofMRI, these changes can be linked to specific differences in graft-host networks (Figure 1(b))—not only in terms of spatial activation, but also in terms of the temporal pattern of causally driven fMRI activity and its polarity. For instance, stimulating the graft at one frequency may drive an increase in firing at a downstream region, while stimulating the graft at another frequency may drive a decrease in firing. Frequency-dependent circuit behavior like this already occurs in the naïve brain [47, 50], but it has not been possible with previous methods to provide this level of detail when characterizing graft-host functional integration. ofMRI allows such responses to be detected across the brain [47], which may prove useful in identifying the neuronal mechanisms that underlie the efficacy or inefficacy of various stimulation paradigms in promoting functional recovery. Note that while the millisecond-timescale precision offered by optogenetics is much faster than the actual rate of BOLD signal changes, which occur on the order of seconds, the spatial location of activation and the general profile of the evoked responses (e.g., polarity, amplitude, and duration) still convey important information dependent on the millisecond precision of optogenetic control.

In addition to varying the temporal pattern of graft control, optogenetics is uniquely suited to probe specific graft-host projections. By using transduction techniques that offer axonal transduction [27, 51–53], light delivery at a specific projection site (away from the somata) can provide control over which graft-host connection is perturbed. Given the many regions that may receive axonal projections from engrafted neurons, this type of characterization will be especially important in advancing our understanding of graft-host integration. For example, in one study that reported functional improvements upon human iPSC transplantation to the striatum of Parkinsonian rodents, engrafted neurons sent axons throughout the basal ganglia, cortex, thalamus, and white matter tracts [54]. When engraftment results in broad structural integration like this, it is virtually impossible to know which connections are essential for functional recovery to occur. However, by selectively and reversibly exciting or inhibiting certain projections and evaluating its effect on recovery, scientists can begin to disentangle these complex circuits (Figure 1(c)). Projection-specific control of graft activity can also be combined with ofMRI to visualize the whole-brain influence of these precise perturbations.

Given the vast parameter space when choosing a stimulation paradigm (including frequency, pulse width, periodic or aperiodic stimulation, excitation or inhibition, and projection-specific targeting), detailed characterization of graft-host circuit function can be a technically arduous task. The development of real-time ofMRI is expected to accelerate the speed at which these parameter spaces can be searched [46, 55, 56]. Closed-loop ofMRI, a platform for updating stimulation parameters based on evoked responses in real time, is also actively being developed and can in principle be applied to more rigorously interrogate causal graft-host interactions [57–59].

In summary, the combination of optogenetics with stem cell biology is poised to address long-standing questions in regenerative medicine of the central nervous system. The specific targeting afforded by optogenetics, as well as its temporally precise and reversible nature, has already enabled the causal influence of graft activity on behavior to be measured in various models of disease. Combining these studies with ofMRI to visualize corresponding graft-host interactions will be an important step in understanding how neural circuit repair underlies functional recovery. Using optogenetics and ofMRI to dissect the role of distinct firing patterns and graft-host projections will also provide novel insight into the graft-host integration process. Such advances may in turn accelerate the development and optimization of various stem cell-based therapies.
